# Material insights of HfO_2_-based integrated 1-transistor-1-resistor resistive random access memory devices processed by batch atomic layer deposition

**DOI:** 10.1038/srep28155

**Published:** 2016-06-17

**Authors:** Gang Niu, Hee-Dong Kim, Robin Roelofs, Eduardo Perez, Markus Andreas Schubert, Peter Zaumseil, Ioan Costina, Christian Wenger

**Affiliations:** 1 Electronic Materials Research Laboratory, Key Laboratory of the Ministry of Education & International Center for Dielectric Research, Xi’an Jiaotong University, Xi’an 710049, China; 2 IHP GmbH/Leibniz-Institut für innovative Mikroelektronik, Im Technologiepark 25, Frankfurt (Oder) 15236, Germany; 3 Department of Electronics, Information & Communication Engineering, Sejong University, Neungdong-ro 209, Gwangjin-gu, Seoul 143-747, Korea; 4 ASM, Kapeldreef 75, Leuven 3001, Belgium

## Abstract

With the continuous scaling of resistive random access memory (RRAM) devices, in-depth understanding of the physical mechanism and the material issues, particularly by directly studying integrated cells, become more and more important to further improve the device performances. In this work, HfO_2_-based integrated 1-transistor-1-resistor (1T1R) RRAM devices were processed in a standard 0.25 μm complementary-metal-oxide-semiconductor (CMOS) process line, using a batch atomic layer deposition (ALD) tool, which is particularly designed for mass production. We demonstrate a systematic study on TiN/Ti/HfO_2_/TiN/Si RRAM devices to correlate key material factors (nano-crystallites and carbon impurities) with the filament type resistive switching (RS) behaviours. The augmentation of the nano-crystallites density in the film increases the forming voltage of devices and its variation. Carbon residues in HfO_2_ films turn out to be an even more significant factor strongly impacting the RS behaviour. A relatively higher deposition temperature of 300 °C dramatically reduces the residual carbon concentration, thus leading to enhanced RS performances of devices, including lower power consumption, better endurance and higher reliability. Such thorough understanding on physical mechanism of RS and the correlation between material and device performances will facilitate the realization of high density and reliable embedded RRAM devices with low power consumption.

Future embedded non-volatile memories require higher density, lower power consumption, higher speed and better scalability^1^. Resistive random access memories (RRAM) with 1 transistor-1 resistor (1T1R) architecture emerge recently as one of the most promising candidates to fulfil these requirements^2^,^3^. Moreover, the RRAM technology is also of great interest for different system-on-chip (SoC) applications such as wireless sensor networks (WSNs) and medical health care devices, considering that RRAM allows to largely reduce the “standby” power dissipation in the sensor node^4^,^5^. In 1T1R RRAM devices, the transistor is used to limit the pulse current in order to avoid the hard breakdown (HBD) in the resistor, which consists of a metal-insulator-metal (MIM) structure for resistive switching (RS). Among various candidates for the active insulator layer in MIM structure^6^, hafnia (HfO_2_) widely attracts attention because of its compatibility with the current semiconductor fabrication process. Since 2007, it has been selected as the “standard” gate dielectric material in modern complementary-metal-oxide-semiconductor (CMOS) transistors^7^. Many efforts have been made to realize fab friendly HfO_2_ RRAM using atomic layer deposition (ALD), which distinguishes itself from other counterparts thanks to its extraordinary good step coverage, the precise control of atomically specified film thickness and the high film quality^8^. Particularly, the batch ALD technique with reduced cost per wafer has been developed for future mass production of related devices^9^. Despite of a bunch of detailed studies focusing on unveiling the RS mechanism (normally on μm-size devices^10^,^11^) and on attempting to improve the cell-performances (with different methods like doping^12^,^13^,^14^,^15^, filament confinement^16^,^17^ and bilayers^18^,^19^,^20^,^21^ etc.), a fundamental but indispensable study correlating the material properties (e.g. the crystallinity and the carbon impurity, etc.) and device performances, particularly for integrated RRAM devices in nm scale, is still missing.

In this work, nm-size 1T1R integrated RRAM devices were fabricated in a standard 0.25 μm CMOS process line. The TiN/Ti/HfO_2_/TiN MIM structures, acting as the resistor, were fabricated by depositing HfO_2_ at two different temperatures (150 °C and 300 °C) by employing the batch ALD approach (with 100 process-wafer loading capability) with a metal organic precursor (which is more suitable for the batch ALD process thanks to its liquid form^22^). Both electrical and material properties of devices were systematically studied and correlated. The nm-size HfO_2_-based devices show typical filament-type RS. The higher deposition temperature of 300 °C does not induce significant recrystallization but dramatically reduces the residual carbon concentration in HfO_2_ films. Increased density of nano-crystallites in HfO_2_ slightly affects the forming voltage and its variability of corresponding devices. Carbon impurities inducing trap levels inside the band gap^23^ are believed to interact with oxygen vacancies (V_O_) in the filament area and thus significantly influence RS properties of the devices. The reduced C concentration in the 300 °C devices leads to enhanced RS performances such as lower V_set/reset_, lower power consumption, better endurance and higher reliability etc. The theoretical simulation using the Quantum Point Contact (QPC) model confirms that the 300 °C samples have much more stable confinement of leakage current paths (i.e. filaments).

## Results and Discussion

### Resistive switching properties

**Figure 1:**
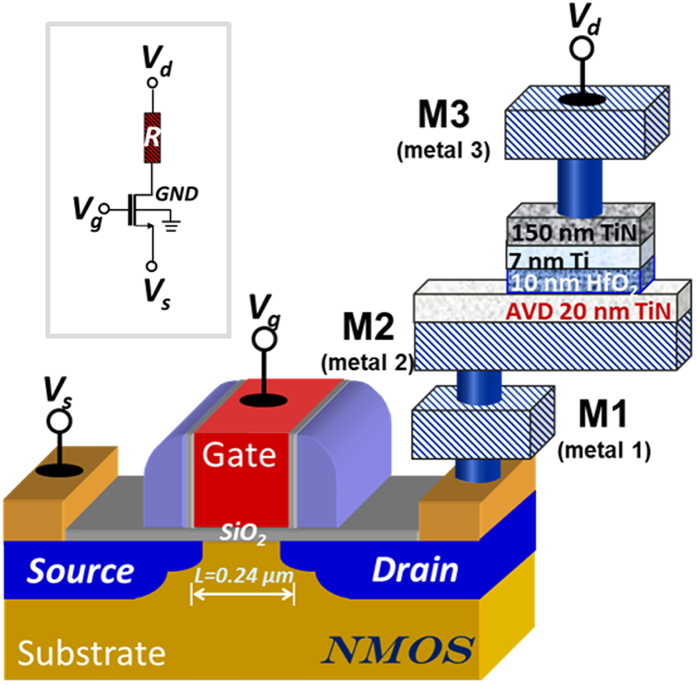
Illustration of HfO_2_-base integrated 1T1R RRAM devices structure, consisting of TiN/HfO_2_/TiN MIM structures and NMOS transistors in series. Inset shows the equivalent circuit.

**Figure 2:**
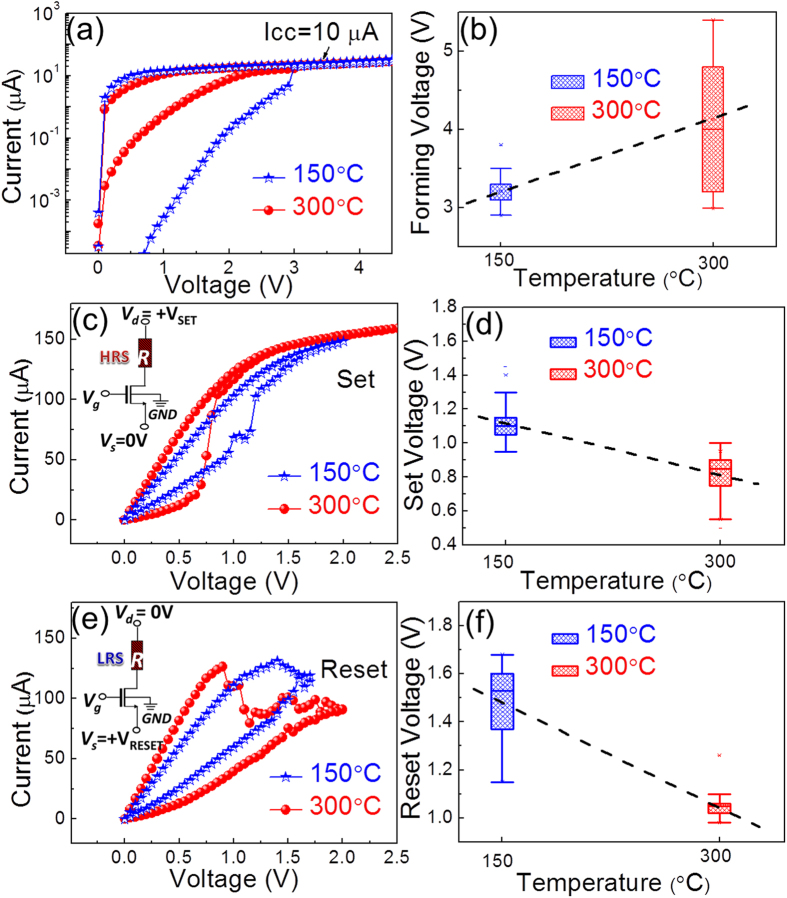
Electrical measurements of 1T1R integrated RRAM devices with HfO_2_ films grown at 150 °C (blue stars) and 300 °C (red circles). The MIM area of the devices is 1000 × 1000 nm^2^. (**a**) Electroforming with I_cc_ = 10 μA; (**b**) V_F_ variation as a function of the HfO_2_ deposition temperature. The dashed line represents the linear fitting result; (**c**) Typical set processes of the devices with the inset demonstrating the equivalent circuits and (**d**) V_set_ variation as a function of the HfO_2_ deposition temperature and the dashed line is a linear fitting; (**e**) Typical reset processes of the devices with the inset demonstrating the equivalent circuits and (**f**) V_reset_ variation as a function of the HfO_2_ deposition temperature and the dashed line is a linear fitting. The boxes in (**b**,**d**) and (**f**) represent 25–75% data range.

Back-end-of-line (BEOL) HfO_2_-based integrated 1T1R RRAM cells were prepared in a standard 0.25 μm CMOS process line. Figure 1 illustrates the final structure of the device. 1T1R devices with five different MIM areas, i.e. 600  × 600 nm^2^, 700 × 700 nm^2^, 800 × 800 nm^2^, 900 × 900 nm^2^ and 1000 × 1000 nm^2^, were processed. The electrical characteristics of 1T1R integrated RRAM devices (using the MIM area of 1000 × 1000 nm^2^) with HfO_2_ films grown at 150 °C and 300 °C have been examined by current-voltage (I-V) measurements. After the electroforming process step, all devices demonstrate resistive switching (RS) behaviours as illustrated in Fig. 2. Figure 2(a) demonstrates the forming process of the 150 °C (blue curve) and the 300 °C (red curve) devices with a common compliance current (I_CC_) of 10 μA (corresponding to a gate voltage of 0.9 V). The forming process was performed by applying a dc sweep on the bit line (BL), i.e., drain line of the select MOS device, up to V_d_ = 4.5 V with a step of 0.05 V (i.e. top injection). To prevent HBD, the current during formation is limited by setting the gate voltage of the select transistor to 0.9 V (word line/WL voltage). It can be observed that during the forward sweep from 0 V to 4.5 V, the current of the pristine 150 °C device is always lower than that of the 300 °C device, indicating a greater initial resistance state (IRS) of the 150 °C sample. Moreover, the 150 °C device demonstrates sharper forming behaviour at 3 V. The variation of the forming voltage (V_F_) as a function of HfO_2_ growth temperature was explored by a statistic study on 20 devices and the result is shown in Fig. 2(b). It can be observed that the V_F_ slightly increases with higher growth temperature (from ~3.2 V to ~4.0 V), which nevertheless results in a much more pronounced V_F_ device-to-device variation (ΔV_F_ varies from ~0.3 V to ~1.8 V).

Figure 2(c) shows the typical set characteristics of the devices. The set operation (see the inset for the equivalent circuit) switches the RRAM cell from a high-resistive state (HRS or OFF state) to a low-resistive state (LRS or ON state) by sweeping V_d_ up to 2.5 V whereas applying 0 V to the source line voltage (V_s_) with V_g_ = 3 V. Figure 2(d) demonstrates the set voltage (V_set_) variation versus HfO_2_ growth temperature. Figure 2(e) shows the typical reset characteristics of devices. The reset operation (see inset for the equivalent circuit) switches the cell from ON state to the OFF state by sweeping V_s_ up to 2 V, whereas V_d_ = 0 and V_g_ = 3 V. Figure 2(f) demonstrates the reset voltage (V_reset_) variation versus HfO_2_ growth temperature. Obviously, one can observe from Fig. 2(c–f) that compared to the 150 °C devices, the 300 °C devices show enhanced RS properties because they possess 1) greater ON/OFF ratios and 2) lower set and reset voltages, reducing the power consumption.

It is well-known that during the electroforming process of oxide-based RRAM devices, the oxide layer undergoes certain nano-morphological modifications associated with the formation of oxygen vacancies (V_O_)^24^,^25^,^26^. Furthermore, the mechanism of V_O_ (or O atoms) movement usually plays a dominant role in determining the RS properties of RRAM devices^10^,^27^. Therefore the observed difference of the 150 °C and the 300 °C devices in their forming and RS behaviour must be closely connected to the RS mechanism and their materials properties, which will be discussed in detail in the following sections.

**Figure 3:**
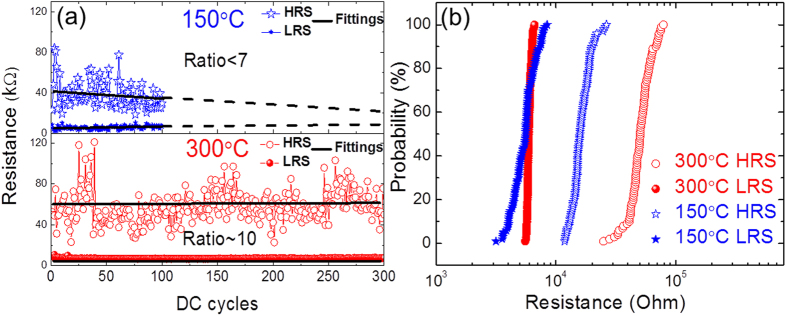
(**a**) The resistance states (at V = 0.2 V of reset) versus dc cycles of 1T1R integrated RRAM devices with HfO_2_ films grown at 150 °C (blue stars) and 300 °C (red circles), respectively; black solid lines show the corresponding linear fit results and the dashed lines are for guiding eyes. (**b**) Probability of resistance states of the 150 °C (blue stars) and the 300 °C (red circles) devices. Empty and full symbols denote HRS and LRS, respectively.

To explore the endurance of the HfO_2_ based 1T1R integrated RRAM devices, dc cycling studies were performed, as shown in Fig. 3. Figure 3(a) shows the resistance (taken from the reset process at V = 0.2 V) evolution with dc cycles for the 150 °C (top panel) and the 300 °C (bottom panel) devices. The straight lines are the corresponding linear fitting results. The 300 °C device shows an ON/OFF ratio of about 10, which is larger than that of the 150 °C device. This confirms the observation by RS loops, as already shown in Fig. 2. Moreover, it is evident that the ON/OFF ratio of the 150 °C device gradually decreases with cycles and the RS “window” continuously becomes smaller. In the contrary, the 300 °C device shows stable endurance behaviour with a constant ON/OFF ratio up to 300 cycles. Interestingly, for both devices the HRS states (empty stars and circles) show higher variations compared to the LRS states (full stars and circles). More details are illustrated in Fig. 3(b), plotting the probability of resistance states. It is confirmed that the LRS with higher slopes (solid symbols) is more stable than the HRS (empty symbols). In particular the LRS of the 300 °C device remains almost unchanged, demonstrating an excellent reliability. Regarding the variation of the HRS, it is related to the RS physical mechanism.

**Figure 4:**
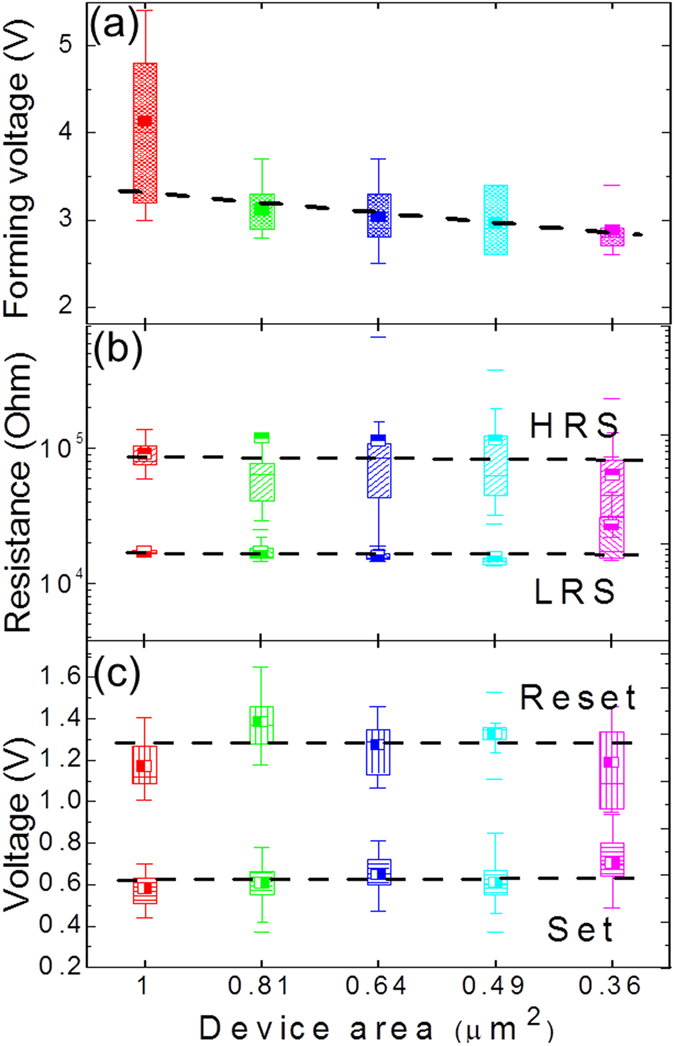
Forming voltage, resistance states and switching voltages (set/reset) versus device area (from 1000 × 1000 nm^2^ to 600 × 600 nm^2^) for the 300 °C devices. The boxes represent 25–75% data range. Black dashed lines are corresponding fittings.

In order to clarify the RS mechanism of such nm-size HfO_2_ based 1T1R integrated RRAM devices, device area dependent I-V measurements have been carried out on the 300 °C devices and the results are shown in Fig. 4. According to detailed reviews by Waser and Sawa *et al.*^1^,^26^,^28^, two main categories of physical models of RS can be classified, i.e. interface type, which is related to the modification of the metal/oxide interface barrier height (thus RS is cell area dependent)^28^ and filament type, the RS of which is dominated by the formation and rupture of local conductive paths inside the oxide bulk (thus RS is cell area independent)^29^. On one hand, it is widely accepted that HfO_2_ based RRAM devices demonstrate V_O_-related filament type mechanism and many studies show experimental observation of the conductive filaments (CFs)^30^,^31^. It is noted here that the CFs in HfO_2_ based devices could also consist of cations^32^ (but specified electrodes are required, in general Cu^2+^) rather than V_O_. Such so-called conductive bridge RAM (CBRAM) is out of the scope of the current work. On the other hand, even for the filament type devices, it is found that the interface details are of great significance^33^,^34^ and different electrodes cause different RS behaviour^24^,^35^. Particularly, TiN or Ti electrodes normally serve as oxygen reservoirs, which strongly influence the V_O_ formation in HfO_2_ and thus the device performance. Our prior studies clarified that the Ti inserting layer is indispensable to achieve good RS behaviour of HfO_2_ based 1T1R integrated RRAM devices and its thickness plays an important role in realizing high RS performance (here an optimized thickness of 7 nm was used)^36^. Furthermore, in-operando hard X-ray photoemission spectroscopy (HAXPES) revealed that an oxygen atom exchange occurs between Ti and HfO_2_ layer during RS processes, which corresponds also to the interface barrier modification^37^,^38^. Therefore, it is necessary to clarify the RS mechanism in nm-sized TiN/Ti/HfO_2_/TiN 1T1R integrated devices, particularly considering that devices areas are comparable with the reported CFs size ranging from a few tens of nanometers to several hundreds of nanometers^27^,^30^,^31^.

Figure 4(a) shows the V_F_ dependence on the device area. It reveals a decrease of V_F_ value scattering and a slight V_F_ reduction when the device area decreases from 1 to 0.36 μm^2^. These behaviours can be attributed to the Ti layer oxidation during the forming process, which helps the formation of V_O_-related filaments in HfO_2_ layer. Many authors pointed out that Ti serves as an excellent oxygen reservoir because the formation of TiO_x_ is easier than HfO_x_^39^,^40^. Ti is oxidized by taking the oxygen atoms from HfO_2_ once the external electric field provides enough energy higher than the reaction activation energy. In cells with smaller Ti/HfO_2_ interface area, less material inhomogeneity (thus smaller V_F_ value scattering) appears and less energy (thus smaller forming voltage) is required to reach the activation energy of Ti oxidation. Moreover, the decrease of V_F_ variation with the reducing device area indicates probably a local area feature of the forming process. This is immediately confirmed by HRS/LRS dependence of device area, as shown in Fig. 4(b). Evidently, the LRS is almost unaffected by the variation of the cell area. The HRS does not show either a clear modification despite of relatively higher error bars. Figure 4(c) further shows that V_set_ and V_reset_ do not vary with the device area. These results evidently confirm that the filament mechanism dominants the RS behaviour of nm-sized HfO_2_ RRAM devices.

After understanding the filament type of our devices, a physics-based analytical quantum point contact (QPC) model was employed to examine the experimental I-V curves in order to unveil more details of the device endurance. Briefly, QPC model assumes that the current flows through a filamentary path between two electron reservoirs in both HRS and LRS states. Then, based on Landauer theory, one can obtain an expression of the conducting current represented by the quantum transmission probability, which is determined by the height and the width of a potential barrier with a parabolic shape. In the HRS, the barrier top is well above the energy window of the injected electrons while in the LRS, the barrier does not impact at all^41^. QPC model has been successfully applied to fit I-V curves of several RS systems including atomic vapour deposited HfO_2_ 1T1R RRAM devices, which highlights again the filament type of such devices^2^,^32^,^42^. Here we focus on the endurance studies. In QPC model, the low resistance current I_LRS_ reads:


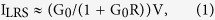


where G_0_ = 2e^2^/h = (12.9 kΩ)^−1^ is the quantum conductance unit; R is the resistance to be fitted for the I-V curve of each cycle and V is the applied voltage. We define G = 1/(1 + G_0_R) which includes the fitted parameter R varied for each RS. Thus the normalized conductance G/G_0_ could indicate the stability of the confinement path (i.e. the filament) in the MIM structure.

**Figure 5:**
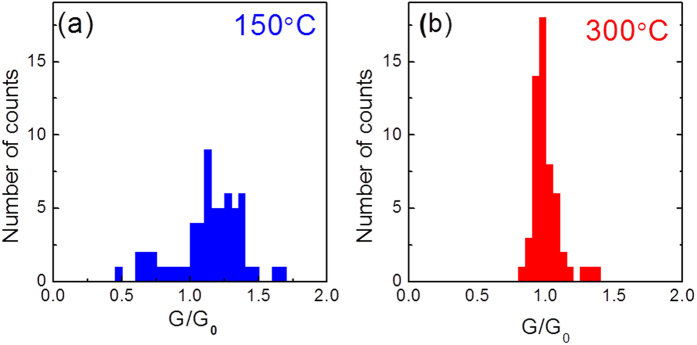
Distribution of the low resistance current values measured during the cycling processes in units of G_0_ for the HfO_2_ based 1T1R devices, deposited at (**a**) 150 °C and (**b**) 300 °C.

Figure 5(a,b) show the distributions of the normalized conductance G/G_0_ for the 150 °C and the 300 °C devices, respectively. They were obtained from the QPC fittings for LRS currents extracted from the similar measurements shown in Fig. 3. It can be seen in Fig. 5(a) that the G/G_0_ of the 150 °C device averagely distributes in a large range from 0.5 to 1.6 despite of a relatively larger intensity at the interval of [1.0, 1.4]. This reveals that the 150 °C device tends to form instable confinement paths (filaments) with large fluctuations, which could be caused by oxygen vacancy transport and inelastic electron trapping and de-trapping processes. These fluctuations decrease the “window” between the LRS and HRS after tens of cycling processes (see Fig. 3(a)). In the contrary, the G/G_0_ distribution of the 300 °C device in Fig. 5(b) represents a very sharp feature with almost all counts locating at the value of 1.0, namely, it is related to a stable confinement path (filament) formed at G_0_. This higher stability of the conductive path of the 300 °C device clearly clarifies the reason for its better endurance compared to that of the 150 °C device (see Fig. 3(a)).

### Material properties and discussion

In order to understand the underlying physical mechanism of the different RS properties in devices induced by the HfO_2_ growth temperatures, 10 nm thick HfO_2_ films were grown by ALD on planar TiN/Si substrates under precisely same conditions as for the 1T1R integrated devices and their properties were explored in detail.

**Figure 6:**
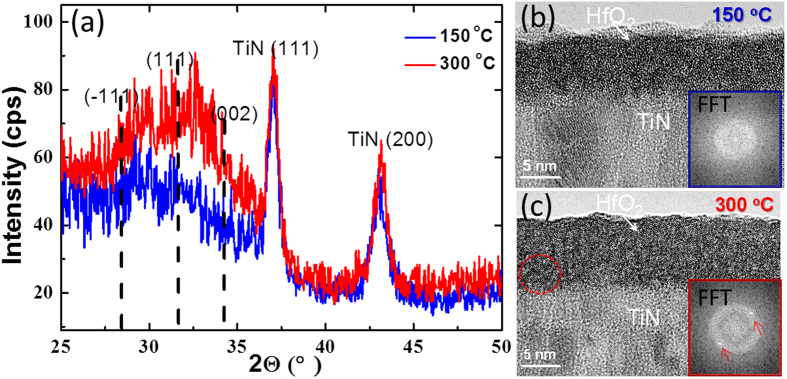
(**a**) Grazing incidence XRD measurements on HfO_2_ films grown at 150 °C (blue) and 300 °C (red) by ALD deposition. The dashed lines show the positions for different orientations of the monoclinic HfO_2_ lattices. HRTEM images of the same ALD HfO_2_ films grown at (**b**) 150 °C and (**c**) 300 °C and insets show FFT patterns. The FFT pattern for the 300 °C film was taken on the circled region where a nano-crystallite is found.

One of the most important film properties which probably influence the RS properties of the device consists in the film crystallinity. Therefore both X-ray diffraction (XRD) and high-resolution transmission electron microscopy (HRTEM) methods were employed to investigate this issue. Figure 6(a) shows grazing incidence XRD (GIXRD) measurements performed at 1° angle of incidence around the TiN (200) Bragg reflection. It is known that the large scale recrystallization of HfO_2_ films occurs above ~370 °C^53^,^44^, below which HfO_2_ remains amorphous. Specular out-of-plane XRD measurements (not shown) do not show any HfO_2_ related diffraction peak. Here GIXRD was utilized, which provides a much higher sensitivity for detecting the possible presence of randomly distributed nm-scale crystallites in the 10 nm-thick amorphous HfO_2_ films. For both films the XRD patterns are dominated by two sharp TiN peaks, i.e. (111) at 2Θ = 36.9° and (200) at 2Θ = 42.6°, which are related to the polycrystalline cubic TiN structure of the substrate. No well-defined HfO_2_ related diffraction peak is observed for both samples, demonstrating that no large-scale recrystallization occurs in both samples. However, a wide swell region ranges from 2Θ~28.3° to 2Θ~34.2° appears in both patterns of the 150 °C and the 300 °C samples, indicating the presence of randomly distributed nm-scale crystallites. The dashed lines denote the reflection positions of (−111) at 2Θ = 28.3°, (111) at 2Θ = 31.6° and (002) at 2Θ = 34.2° of HfO_2_ monoclinic lattices. The swell regions well locate between (−111) and (002) positions. Moreover, the swell region of the 300 °C sample has a slightly higher intensity indicating an increase of nm-size crystallites. HRTEM measurements were therefore performed to possibly visualize the crystallites, as shown in Fig. 6(b,c) for the 150 °C and the 300 °C samples, respectively. For the 150 °C sample, no crystallite was found in the detected region. That is the reason why Fast Fourier Transformation (FFT) pattern (Fig. 6(b) inset) extracted from an arbitrary part of the HfO_2_ film shows only a diffused halo feature. While for the 300 °C sample, FFT patterns extracted from most parts of the film in Fig. 6(c) show the same pattern as the inset of Fig. 6(b), indicating that the overwhelming part of the 300 °C film remains amorphous. However, one can find a single crystallite with diameter of ~2 nm in the circled region and thus the FFT pattern (Fig. 6(c) inset) extracted from this part shows both diffused halo and dots (marked by arrows).

Let us now consider the impact of the film crystallinity on the RS properties shown in the last section. Firstly, it is known that polycrystalline grain boundaries (GBs) could serve as favourable conductive paths due to their oxygen deficient feature, which lead to higher conductivity of the HfO_2_ films^45^,^46^,^47^. Recently, conductive atomic force microscopy (CAFM) studies have provided solid proofs that, under nanoscale electrical stress, the leakage currents through GBs are higher than those through the grains in HfO_2_ films^48^. Meanwhile, the electrical breakdown voltages on GBs are much lower than those on grains (nanocrystals), leading to the formation of CFs at GBs that are responsible for the repeatable RS behaviour observed in Hf based oxide films^49^. Compared to the 150 °C sample, the 300 °C sample remains amorphous but shows an increasing crystallite density. This explains its smaller IRS before forming, as observed in Fig. 2(a). Moreover, more inhomogeneous feature in the 300 °C sample leads to a larger V_F_ variation, as shown in Fig. 2(b). Thirdly, according to our prior theoretical and experimental studies on the Ti/HfO_2_ interface, Ti can scavenge more O from amorphous HfO_2_ films compared to polycrystalline ones^50^. Therefore, the increasing number of nm-size crystallites yields higher average V_F_.

**Figure 7:**
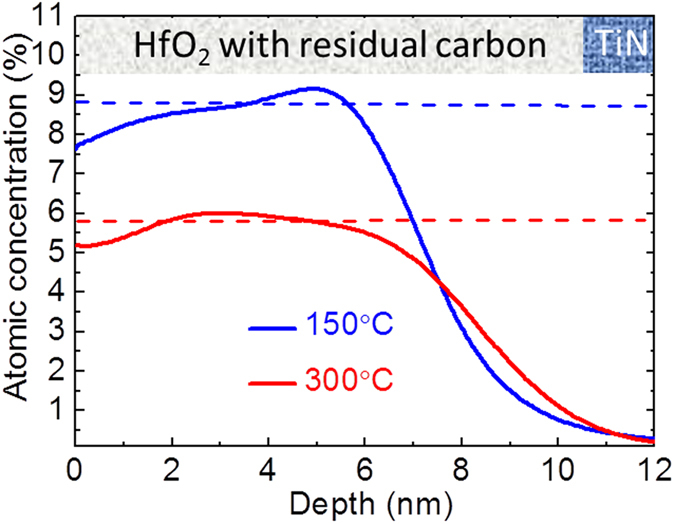
Tof-SIMS depth profile showing the relative atomic concentration of residual carbon in HfO_2_ films deposited at 150 °C (blue) and 300 °C (red) as a function of the detecting depth. The dashed lines mark the average values of C concentration for both samples.

These considerations demonstrate that the difference in forming process of both devices can be well attributed to the film crystallinity. However, such arguments meet difficulties when one tries to clarify the difference of the V_set_/V_reset_ and the endurance behaviour of the devices fabricated at different temperatures, because there is no clear proof, demonstrating that the filaments formed in HfO_2_ films with slightly different crystallinity possess different behaviour. Meanwhile, solid evidences have been reported for the inevitable presence of residual impurities like carbon or chlorine (depending on the precursors) in ALD grown HfO_2_ films and their significant impact on the electrical properties of HfO_2_ such as carrier mobility and reliability in metal-oxide-semiconductor (MOS) devices^51^,^52^. Particularly, the HBD of MOS and/or MIM devices has been correlated with the high C concentration in the HfO_2_ layer^51^,^52^,^53^. In this study, carbon related TEMAH precursor has been utilized due to its liquid form being suitable for the batch ALD process, therefore, the residual carbon atomic concentration in both films grown at 150 °C and 300 °C were explored. Time-of-flight secondary ion mass spectrometry (Tof-SIMS) method was employed to determine the carbon content because it provides a very sensitive detection range up to 10^18^ atoms/cm^3^ (~10 ppm). Figure 7 displays the Tof-SIMS depth profile for carbon residues in ALD HfO_2_ films as a function of the film depth, in which the atomic concentration values were determined by the estimation from sputtering X-ray photoelectron spectroscopy (XPS) measurements (see Fig. S1 in Supporting Information). It can be seen that the HfO_2_ film grown at the lower temperature of 150 °C (blue curve) reveals an average residual carbon concentration of ~8.9% (marked by a blue dashed line) whereas the higher temperature of 300 °C (red curve) results in a lower one of ~5.9% (marked by a red dashed line) in the HfO_2_ film. The reduction of C residues at higher temperatures in ALD HfO_2_ films originate either from the almost complete decomposition of the Hf[N(CH_3_)(C_2_H_5_)]_4_ precursor or the more complete reaction between the precursor and oxidants during the deposition.

C defects can exist as substitutional and interstitial ones. Cho *et al.*^51^,^52^ clarified using density functional theory approach that, the interstitial defect is favoured under the oxygen-rich ambience whereas the substitutional form is more stable within the oxygen-deficient environment. More importantly, Choi *et al.*^23^ further pointed out that carbon atoms which induce trap levels inside the band gap can form a permanently conducting path once they percolate. This indicates that, similarly as V_O_ which forms filaments contributing to a “soft” breakdown in RRAM devices, C residues can also form filaments which results in, however, “hard” breakdown. It is worth noting that during the forming process in RRAM devices, the initial formed filament should still be based on V_O_ instead of C atoms due to the higher mobility of oxygen ions and the easier formation of V_O_ thanks to the reservoir effect of the Ti metal^36^,^39^.

Following this scenario, it is probable that once the V_O_-based filament is formed in the HfO_2_ film, C residues that are initially homogeneously distributed in the film tend to shift into the V_O_ sites in the filament due to its oxygen-deficient feature. Considering that the reset process in RRAM devices is based on the migration of O cations and its refilling into V_O_ sites^54^, the C filling in V_O_ sites (C can form complexes with V_O_^55^) and the formation of the more stable the Hf-C (with dissociation energy of 379 kcal/mole^56^ compared to a much smaller value of 184 kcal/mole of Hf-O bond^57^) will impede the reset process, i.e. it requires higher energy to dissociate the filament. This is precisely what we observed in Fig. 1(c–f). The 150 °C sample with higher C concentration requires higher V_set_ (~1.1 V) and V_reset_ (~1.5 V) to switch the device while V_set_ and V_reset_ of the 300 °C sample are only ~0.8 V and ~1.0 V respectively. In addition, more C atoms in V_O_-filament of the 150 °C device influence the leakage behaviour of its HRS state, i.e. they yield higher currents in HRS thus a smaller ON/OFF ratio (~7) compared to that of the 300 °C device (~10).

The inferior endurance of the 150 °C device (Fig. 3(a)) can also be ascribed to its higher C residual concentration. It is likely that the 150 °C device (~8.9% C residue), with the cycles (continuous electrical stress), more and more C atoms are “filled” into V_O_ sites thus the filament transforms from a V_O_-dominant to a C-dominant feature with enhanced leakage current^55^ and finally a pure C filament forms leading to a hard breakdown. While for the 300 °C device (~5.9% C residue), the C concentration does not exceed the critical value for the formation of a “percolation path” therefore much better endurance and reliability (Figs 3 and 5) were realized. Our prior in-operando HAXPES study of the Ti/HfO_2_/TiN system also revealed a clear carbon segregation towards the Ti/HfO_2_ interface, which is a reservoir layer of V_O_, with RS cycles^37^,^38^,^58^, confirming the interaction between C and V_O_ thus the formation of C-V_O_ complexes with RS cycles.

**Figure 8:**
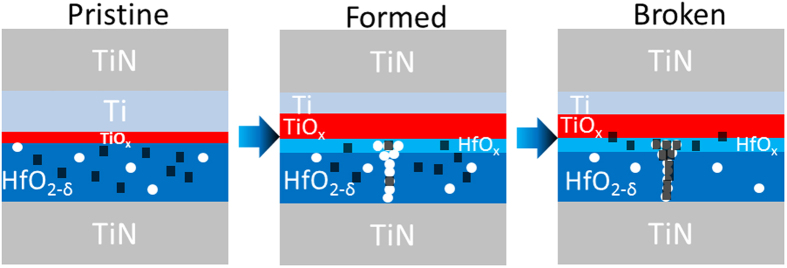
Schematic illustration of the C-related hard breakdown mechanism in HfO_2_ based RRAM devices. From left to right: the pristine state after device processing, the electroformed state and the broken state of the device. Black squares represent the carbon impurities and the oxygen vacancies are denoted by white circles.

Figure 8 illustrates this C residue related hard breakdown mechanism in the 150 °C device. In the pristine state, carbon impurities (black squares) are randomly distributed in the oxide film. It is noted here that due to a sintering process for 1T1R devices, consisting of a thermal annealing at 400 °C^2^,^36^, the Ti layer was already partly oxidized and V_O_ appear in the HfO_2_ film which is thus denoted as HfO_2−δ._ After the forming process, Ti (as an oxygen reservoir) absorbs even more O ions from HfO_2−δ_ film under the externally applied electric field and thus an oxygen deficient HfO_x_ layer as well as a V_O_-filament (white circles) forms. At the same time, a few C atoms shift, due to their high concentration, to HfO_x_ layer as well as V_O_ sites in the filament. With cycles, more and more C-V_O_ complexes form and a filament consisting of mainly C eventually forms which leads to the hard breakdown of the device.

## Conclusion

In conclusion, nm-size HfO_2_-based 1T1R integrated RRAM devices were fabricated in a standard 0.25 μm CMOS process line by employing the batch ALD tool designed for mass production. We demonstrated material insights for the RS properties of devices and correlated the key material properties, including the crystallinity and the residual C concentration, with the device performances. Cell-area dependent studies were performed, indicating their filament-type RS feature even in nm-size, despite a strong impact of the interface on the forming process. A relatively higher deposition temperature of 300 °C results in a lower IRS and a slightly higher V_F_ due to the increasing density of nm-size crystallites in the mainly amorphous bulk structure of the film. Moreover, the residual C impurity, which induces traps in the bandgap of HfO_2_ (similarly as V_O_) and could interact with V_O_^23^,^51^,^52^, imposes significant impact on the RS properties of the devices. Thanks to the reduction of the C impurity concentration, the 300 °C device displays lower power consumption with smaller V_set_ and V_reset_, improved endurance and a better filament control thus higher reliability. The device performance-material property correlation improves the understanding of the underlying physics of the resistive switching in MIM structure and will certainly help to optimize the fabrication process of the 1T1R embedded RRAM cells thus enhancing their performances for the future high density SoC applications such as wireless sensor networks and medical health care devices.

## Methods

### Fabrication of HfO_2_ based 1T1R RRAM cells

A standard 0.25 μm CMOS process line was employed. Figure 1 illustrates the final structure of the device. Firstly, the NMOS transistors were processed with width (W) of 1.14 μm and length (L) of 0.24 μm. The resistive switching cell was then placed between the metallization levels 2 and 3. In order to reduce the surface roughness of the bottom electrode, a 20 nm-thick TiN layer was additionally deposited by atomic vapour deposition (AVD). 10 nm HfO_2_ films were grown at 150 °C and 300 °C in an ASM A412™ batch ALD system (with 100-wafer load capability, specially designed for mass production) using a metal organic TEMAH (tetrakis (ethylmethylamino)hafnium, Hf[N(CH_3_)(C_2_H_5_)]_4_) precursor^22^. Finally, HfO_2_ was capped by a 7 nm ionized metal plasma (IMP) Ti layer then a 150 nm PVD TiN^59^ layer.

### Electrical characterization and analysis of the RRAM cells

The electrical properties of the memory cells were measured in a Cascade PA200 Semi-automatic Probe System, and the current-voltage (I-V) curves were collected by using a Keithley 4200 semiconductor parameter analyser. I-V characteristics were theoretically simulated by a physics-based analytical QPC model^41^,^60^.

### Film crystallinity and thickness measurements

the crystalline properties of the HfO_2_ films were examined by X-ray diffraction (XRD) under grazing incidence conditions using a Rigaku Smartlab diffractometer with a 9 kW rotating anode (Cu Kα1, λ = 1.5406 Å) and microscopically by high resolution transmission electron microscopy (HRTEM) using a FEI Tecnai Osiris equipment operated at 200 kV. The film thicknesses were also determined by TEM measurements.

### Residual carbon concentration measurements

The atomic concentration of residual carbon in the HfO_2_ films was characterized by both sputtering X-ray photoelectron spectroscopy (XPS) with Al Kα excitation energy (1486.6 eV) using a PHI Versa Probe II Scanning XPS Microprobe system and time-of-flight secondary ion mass spectrometry (Tof-SIMS) using an IONTOF TOF-SIMS 5 equipment with an Cs sputtering beam (500 eV) and a Bi_1_ analysis beam (25 keV).

## Additional Information

**How to cite this article**: Niu, G. *et al.* Material insights of HfO_2_-based integrated 1-transistor-1-resistor resistive random access memory devices processed by batch atomic layer deposition. *Sci. Rep.* **6**, 28155; doi: 10.1038/srep28155 (2016).

## Supplementary Material

Supplementary Information
