# Supplementary material for: Material insights of HfO2-based integrated 1-transistor-1-resistor resistive random access memory devices processed by batch atomic layer deposition
Source: Sci Rep. 2016 Jun 17;6:28155. doi: 10.1038/srep28155 (PMC4911574; doi:10.1038/srep28155)
Supplement: Supplementary Information [file srep28155-s1.doc]

Supplementary Information

Material insights of HfO_2_-based integrated 1-transistor-1-resistor resistive random access memory devices processed by batch atomic layer deposition

Gang Niu^1,2*^, Hee-Dong Kim^3^, Robin Roelofs^4^, Eduardo Perez^2*^, Markus Andreas Schubert^2^, Peter Zaumseil^2^, Ioan Costina^2^, Christian Wenger^2^

^1^Electronic Materials Research Laboratory, Key Laboratory of the Ministry of Education & International Center for Dielectric Research, Xi'an Jiaotong University, Xi'an 710049, China.

^2^IHP GmbH/Leibniz-Institut für innovative Mikroelektronik, Im Technologiepark 25, Frankfurt (Oder) 15236, Germany.

^3^Department of Electronics, Information & Communication Engineering, Sejong University, Neungdong-ro 209, Gwangjin-gu, Seoul 143-747, Korea

^4^ASM, Kapeldreef 75, Leuven 3001, Belgium

*Corresponding author: [gangniu@mail.xjtu.edu.cn](mailto:gangniu@mail.xjtu.edu.cn)

*Corresponding author: [perez@ihp-microelectronics.com](mailto:perez@ihp-microelectronics.com)


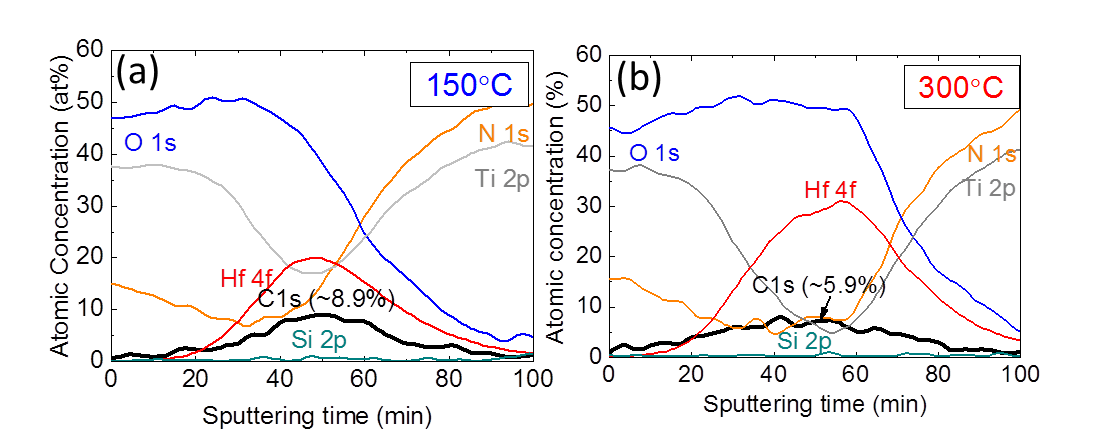


Figure S1. Atomic concentration depth profiles as a function of the sputtering time (in the unit of minute) for the (a) 150°C and (b) 300°C devices consisting of the Ti/HfO_2_/TiN/Si stack. The atomic concentrations were determined by quantitatively analyzing the XPS peaks of O 1s (blue), Hf 4f (red), C 1s (black), Ti 2p (grey), N 1s (orange) and Si 2p (green). The extracted C concentration of the 150°C and the 300°C devices are ~8.9% and ~5.9%, respectively, which were used as reference values for Tof-SIMS results shown in Figure 7.

The chemical depth profiling of different elements including residual carbon in the Ti/HfO_2_/TiN stack were characterized by sputtering X-ray photoemission spectroscopy (XPS) with Al Kα excitation energy (1486.6eV) using a PHI Versa Probe II Scanning XPS Microprobe system.
